# Síndrome de *burnout* en odontólogos de centros de salud de Acapulco, México

**DOI:** 10.21142/2523-2754-1102-2023-150

**Published:** 2023-06-29

**Authors:** Tatiana Ciprián Chavelas, Eslin Adame Marroquín, Carlos Alberto Juárez Medel

**Affiliations:** 1 . Dirección General de Salud Municipal de Acapulco. Guerrero, México. tatiana_ciprian09@outlook.es, slin_vlf36@hotmail.com Dirección General de Salud Municipal de Acapulco Guerrero México tatiana_ciprian09@outlook.es slin_vlf36@hotmail.com; 2 Coordinación de Formación y Capacitación del Personal de Salud de la Unidad de Coordinación Nacional Médica. Instituto de Salud para el Bienestar. Guerrero, México. carlos.juarez@insabi.gob.mx Coordinación de Formación y Capacitación del Personal de Salud de la Unidad de Coordinación Nacional Médica Instituto de Salud para el Bienestar Guerrero México carlos.juarez@insabi.gob.mx

**Keywords:** burnout, odontólogos, servicios públicos de salud, centros de salud (DeCS), burnout syndrome, dentists, public health service, ambulatory care facilities (MeSH)

## Abstract

**Introducción:**

El síndrome de *burnout* es el agotamiento profesional resultado de un estrés laboral crónico con un complejo tridimensional caracterizado por agotamiento emocional, despersonalización y realización personal derivadas del trabajo por contacto directo con los pacientes.

**Objetivo:**

Estimar la prevalencia del síndrome de *burnout* severo en odontólogos de centros de salud de Acapulco, México.

**Material y métodos:**

Diseño transversal en una muestra por conveniencia de 80 odontólogos con la aplicación de un cuestionario autoadministrado de 44 ítems durante marzo a mayo del 2022. El instrumento contuvo el Inventario Maslach-Burnout de 22 ítems con respuestas categoriales tipo Likert, que establecieron una puntuación global con la suma de las tres dimensiones. Se aplicó un análisis de regresión logística binaria a partir del punto de corte establecido con el *software* estadístico de SPSS V.24.0.

**Resultados:**

El 50% de los odontólogos presentó *burnout* severo. Se encontró un factor relacionado, el tipo de contratación laboral, en la categoría contrato eventual y por honorarios (p = 0,04).

**Conclusión:**

Deben ejecutarse acciones que mejoren las condiciones de estabilidad laboral en los odontólogos, a través del reconocimiento de su trabajo para mejorar los tipos de contratación en las instituciones de salud del ámbito público.

## Abstract (en)

**Introduction:**

burnout syndrome is professional burnout resulting from chronic work stress with a three-dimensional complex characterized by emotional exhaustion, depersonalization and personal fulfillment derived from work due to direct contact with patients.

**Objective:**

To estimate the frequency of severe burnout syndrome among dentists of Health Centers from Acapulco, Mexico.

**Material and methods:**

Cross-sectional design in a convenience sample of 80 dentists with the application of a self-administered questionnaire of 44 items during march to may 2022. The instrument contained the Maslach-Burnout Inventory of 22 items with Likert-type categorical responses, which established a global score with the sum of the three dimensions. A binary logistic regression analysis was applied based on the cut-off point established with SPSS V.24.0 statistical software.

**Results:**

The 50% of the dentists presented severe burnout. A related factor was found, the type of employment contract, in the category of temporary and fee contract (p=0.04).

**Conclusion:**

Actions should be taken to improve labor stability conditions for dentists, through the recognition of their work, in order to improve the types of contracting in public health institutions.

## INTRODUCCIÓN

La Organización Mundial de la Salud (OMS) define al síndrome de *burnout* (SB) como el agotamiento profesional resultado de un estrés laboral crónico que no ha sido gestionado con éxito. Está incluido en la 11.ª Revisión de la Clasificación Internacional de Enfermedades (CIE-11) como fenómeno ocupacional y no está considerado como afección médica ^1^. La psicóloga social Christina Maslach propuso por primera vez el término en una conferencia de la Asociación Americana de Psicología (APA) en 1976, y cinco años después lo definió como un complejo tridimensional caracterizado por agotamiento emocional, despersonalización y realización personal derivadas del trabajo por contacto directo con clientes o pacientes ^2^.

En 1982, Cherniss definió el *burnout* como un proceso en el que las actitudes y conductas de los profesionales cambian de forman negativa frente al estrés laboral condicionado por factores organizacionales. Los profesionales que tienen mayor vulnerabilidad al *burnout* son aquellos con interacciones humanas de carácter intenso y duradero ^3^. El SB afecta la calidad de vida, la salud mental y pone en riesgo la vida de quienes lo padecen. De acuerdo con la Organización para la Cooperación y Desarrollo Económicos (OCDE), México ocupa el primer lugar en estrés laboral con jornadas de trabajo excesivas y menos días de vacaciones al año en comparación con otros países. El estudio de la Escala Mexicana de Degaste Ocupacional (EMEDO), realizado por la Universidad Nacional Autónoma de México (UNAM), en más de 500 profesionistas entre 25 y 40 años, demostró que el 100% presentó algún grado de estrés y el 60% se encontró en un estado alto, con manifestación de daños físicos ^4^.

En los profesionales de atención primaria de la salud, se ha documentado que al menos dos de cada cinco padecen SB ^5^^,^^6^. A nivel global, se estima que la prevalencia del *burnout* en odontólogos es del 13% y la prevalencia total en las subescalas es del 28% para el agotamiento emocional, el 18% para la despersonalización y el 10% para la realización personal. En cuanto a los niveles de las subescalas, el 25% presenta agotamiento emocional alto; el 18%, despersonalización alta, y el 32%, falta de realización personal alta. Se hace mención que en la práctica odontológica hay una prevalencia considerable del SB, principalmente en la en la dimensión del agotamiento emocional ^7^.

En la práctica odontológica, los cirujanos dentistas están sujetos a la expectativa y constante evaluación del paciente, así como a un desgaste emocional que supone la interacción tratamiento-salud. Además, existen factores predisponentes que hacen que los profesionales de este campo sean susceptibles al desgaste profesional, tales como la sobrecarga laboral, las complicaciones del tratamiento, las consecuencias de la tensión emocional del paciente y, en ocasiones, el ejercicio solitario, puesto la responsabilidad no puede ser compartida y es frecuente la sensación de soledad. Se incorporan la ambigüedad en la relación paciente-profesional, el riesgo económico por las gestiones propias de la empresa y el ejercicio profesional en ambientes cerrados e inseguros ^8^.

El estrés afecta negativamente el funcionamiento físico, mental y emocional de los profesionales odontólogos; es por ello que este personal debe ser consciente de los factores de riesgo y estar motivado para comprenderlos, así como las desventajas de la profesión, a fin de aprender estrategias efectivas para manejarlas ^9^. Cabe destacar que, ante la pandemia ocasionada por la enfermedad del coronavirus de 2019 (COVID-19), se ha visto reflejada la angustia psicológica y el agotamiento en el personal odontológico ^10^. Ante ello, las instituciones sanitarias deben llevar a cabo mejoras estratégicas en los sitios de trabajo que mejoren la convivencia para el personal de odontología. En la región, no hay estudios que analicen la problemática del *burnout* en los profesionistas dentales; por tanto, el propósito de esta investigación fue estimar la prevalencia del SB severo en odontólogos de los centros de salud de Acapulco (Guerrero, México).

## MATERIAL Y MÉTODOS

El protocolo de investigación cumplió con los principios éticos y fue aprobado por la Dirección de Enseñanza de la Jurisdicción Sanitaria 07 de Acapulco (México), así como no generó ningún riesgo para la salud de los encuestados. El jefe jurisdiccional permitió la recolección de la información, de manera que los encuestados contestaran libre y voluntariamente bajo consentimiento informado. La encuesta no recopiló datos sensibles de los participantes, por lo que se garantizó su anonimato. 

El diseño del estudio fue transversal en odontólogos del primer nivel de atención en centros de salud del sector público adscritos a la Jurisdicción Sanitaria 07 de Acapulco, México. Por cuestiones de facilidad, se realizó un muestreo por conveniencia en 86 odontólogos distribuidos en la localidad durante el periodo de marzo a mayo de 2022. Los criterios de inclusión fueron odontólogos habilitados en los servicios. Se excluyó a cuatro odontólogos que desempeñaban otras funciones no relacionadas con la práctica odontológica y se eliminaron dos encuestas incompletas, por lo que la muestra final fue de 80 profesionales. 

El instrumento de medición fue un cuestionario autoadministrado de 44 ítems, diseñado por ronda de expertos ^11^. Se recopiló información sociodemográfica a través de tres ítems: género, edad y estado civil. Para el nivel socioeconómico, se utilizaron cinco ítems: número de cuartos para dormir, tipo de piso, tenencia de computadora, tenencia de celular y acceso a internet en el hogar, cada uno con una determinada puntuación ^12^, además de otro ítem no contable que obtuvo el número de personas dependientes del ingreso del encuestado. La situación laboral incluyó siete ítems: zona del centro de trabajo, tipo de contratación, experiencia laboral, turno laboral, carga horaria laboral semanal, ejercicio en la práctica privada y preocupaciones para el desempeño laboral. Otros seis ítems recopilaron información sobre antecedentes de COVID-19.

Los 22 ítems restantes abordaron el Inventario Maslach-Burnout (MBI) traducido al español, ya validado en personal de salud (alfa de Cronbach del 70%) ^13^^,^^14^ y con respuestas categóricas de Likert. Los encuestados valoraron la intensidad con la que experimentan sus sentimientos en una escala de 7 puntos que va de 0 (nunca) a 6 (todos los días) en las tres dimensiones (agotamiento emocional, despersonalización y realización personal). Se sumaron las puntuaciones de las dimensiones del inventario y se estableció el corte a partir de las puntuaciones ≥ 67 para definir al SB en estadio severo ^15^.

El análisis estadístico de los datos se realizó con el *software* SPSS V.24.0 ^16^. Como primer paso, se efectuó un análisis univariado que obtuvo distribuciones simples de las variables. El SB fue operacionalizado en una variable dicotómica a partir del punto de corte, por lo que se aplicó una regresión logística binaria que estableció la relación entre la variable dependiente (SB severo) y los demás factores, a través de un modelo explicativo con la razón de momios y su intervalo de confianza del 95% como estimadores ^17^. El modelo inicial permitió conocer la influencia de los factores sobre el SB severo, y se eliminaron en cada paso variables con las asociaciones más débiles, mediante el método *backward*, hasta quedar aquellas con nivel de significancia p < 0,05 en el modelo final ^18^.

## RESULTADOS

El 51% (41/80) de los odontólogos fueron hombres y el resto fueron mujeres. La edad osciló entre 27 y 74 años, con una media de 44,15 ± 11,20. Respecto del estado civil, el 46% (37/80) es casado; el 25% (20/80), soltero; el 13% (10/80), en unión libre; otro 13% (10/80), divorciado, y el 3% (3/80), viudo. Sobre el nivel socioeconómico, el 90% (72/80) de los odontólogos se categorizó en el nivel medio, lo que da a entender que la mayoría cuenta con los servicios básicos en el hogar, y el resto fue alto. Un 51% (41/80) de los ingresos de los odontólogos proveen el sustento a una o dos personas en el hogar.

La mayoría de los establecimientos de salud estuvieron en la zona urbana, con el 67% (54/80). El 77% (67/80) tienen estabilidad laboral por contrato de base y un 42% (34/80) cuenta con una antigüedad laboral de 11 a 20 años. Sobre el turno laboral, el 42% (34/80) está en el matutino, con una carga laboral semanal de 41 horas o más (57%; 46/80). El 62% (50/80) cuenta con práctica privada. Entre las principales preocupaciones en el sitio laboral está la COVID-19, con el 41% (33/80), seguida por la falta de insumos para los procedimientos ejecutados, la inseguridad en la zona y la situación financiera. 

Sobre los antecedentes de la COVID-19, el 80% (64/80) ya ha tenido infección por el virus, un 97% (77/80) manifestó que sus familiares lo han padecido y el 71% (57/80) comentó que algún familiar falleció como consecuencia de dicha enfermedad. El 82% (66/80) de los odontólogos mencionaron que atendieron a pacientes con antecedentes de infección de COVID-19 en sus centros laborales. La mayor parte de los odontólogos mencionaron tener su esquema de vacunación completo (98%; 78/80) y solo dos comentaron que, por causas de enfermedad y de tiempo, no lograron completar su esquema. 

Respecto de las dimensiones, en el agotamiento emocional, las puntuaciones oscilaron de 0 a 52 puntos, con una media de 23,30 ±16,38, y un 59% (47/80) se categorizó en alto. En la despersonalización, las puntuaciones oscilaron de 0 a 30 puntos, con una media de 7,19 ±7,16, y el 29% (23/80) se categorizó en alto. En la dimensión de realización personal, las puntuaciones oscilaron de 5 a 48 puntos, con una media de 37,76 ± 10,16, con un 46% (19/80) en criterio alto. Para determinar la prevalencia global del SB, se sumaron las puntuaciones de las tres dimensiones. Los valores oscilaron entre los 17 y 129 puntos, con una media de 68,19 ±26,12 y, al categorizar, el 50% (40/80) de los odontólogos presentó SB severo (Tabla 1).

**Tabla 1:** Parámetro global del síndrome de *burnout*

Parámetro	Categoría	n = 80	%
≤ 33 puntos	Bajo	8	10%
34 a 66 puntos	Moderado	32	40%
≥ 67	Severo	40	50%

Se aplicó un modelo explicativo de regresión logística binaria asociado al SB severo, el cual incluyó los factores del tipo de contratación, el turno laboral, el ejercicio de la práctica privada, la antigüedad, la carga horaria laboral semanal, la situación socioeconómica y los antecedentes de COVID-19. Fueron eliminándose las variables una a una y solo el tipo de contratación laboral resultó ser significativo en el modelo final. Los odontólogos con un tipo de contrato eventual y por honorarios tienen alrededor de tres veces mayor probabilidad de padecer SB severo (p = 0,04). En la Tabla 2 se muestra el modelo final de la regresión logística binaria con la razón de momios y su intervalo de confianza del 95%.

**Tabla 2:** Modelo final de regresión logística binaria

Factores	Categoría	RM	IC95%	p
Tipo de contratación	Contrato	3,68	1,08 - 12,53	0,045*
	Por honorarios	3,89	1,13 - 13,39	0,043*

RM = Razón de momios IC 95% = Intervalo de confianza del 95% p = Nivel de significancia * Significativo

El modelo inicial incluyó las variables tipo de contratación, turno laboral, ejercicio de la práctica privada, antigüedad, carga horaria laboral semanal, situación socioeconómica y antecedentes de COVID-19.

## DISCUSIÓN

Esta investigación estimó la prevalencia del SB severo en odontólogos de los centros de salud adscritos a la Jurisdicción Sanitaria 07 de Acapulco, México. La mitad de los odontólogos se ubicó en esta categoría. El tipo de contratación laboral fue un factor relacionado con el SB severo. 

Entre las limitaciones del estudio estuvo el diseño, ya que, al ser transversal, no establece los criterios de temporalidad entre las variables. Es importante señalar que la encuesta fue llevada a cabo durante la transición de la cuarta y quinta ola de la pandemia, por lo que es posible que las preocupaciones derivadas de la misma hayan impactado en los resultados. Posiblemente exista un sesgo de selección no diferencial, puesto los centros de salud donde laboran los profesionistas tienen diferentes condiciones en cuanto a su localización, y quizá esto tenga un impacto en sus percepciones. Otra limitante fue el muestreo no probabilístico, que imposibilita extrapolar los resultados en los profesionales de odontología de la región. 

En cuanto a las dimensiones, el 59% de los odontólogos mostraron prevalencias altas en el agotamiento emocional, resultados casi similares a los reportados por Orozco *et al*. en odontólogos del Seguro Social Campesino de Chimborazo, Ecuador ^19^. Ogdon-Lebrón *et al*. ^20^ mencionan que al menos seis de cada diez odontólogos presentan puntuaciones bajas en esta dimensión en los servicios de la Región Sanitaria de Asunción, en Paraguay. En el aspecto de la despersonalización, la mayor parte de los odontólogos mantiene cifras bajas, lo cual difiere de lo reportado en odontólogos de Paraguay, donde más de la mitad presenta tendencia alta ^20^. Navarro-Guitart *et al*. ^21^ encontraron que las cifras altas en esta dimensión las padecen menos de la mitad de los odontólogos de Córdoba, en Argentina, lo que es similar a nuestros resultados. En la realización personal, más de la mitad de los odontólogos presentaron puntuaciones de la categoría alta, tal como lo reportado en odontólogos paraguayos ^20^. En otros estudios se ha encontrado mayor prevalencia a puntuaciones bajas ^19^.

En cuanto al global del *burnout*, la mitad de los profesionistas presentaron la categoría de severo. En otros estudios referenciados se encontraron cifras bajas, con un rango del 7% al 31% ^20^^-^^23^. De acuerdo con ciertos factores asociados, la literatura sugiere que los problemas de conciliación laboral y familiar ^22^, el trabajar en el sector público y privado, el tiempo de servicio ^21^, el cargo, la profesión y la institución en la que laboran se asocian con las altas tasas de *burnout* en los odontólogos ^24^. En cuanto a las variables biológicas, como el género, no encontramos diferencias en la prevalencia; sin embargo, un estudio realizado en el Perú documenta que las mujeres tienen mayor propensión al *burnout*^24^.

En nuestro estudio, se encontró que factores implicados al SB fueron el tener un contrato eventual o por honorarios; por lo que es posible que la incertidumbre de no tener estabilidad laboral influya en la presencia de cifras más altas. Además, es importante destacar que la pandemia de COVID-19 provocó una crisis financiera en el área odontológica a nivel mundial, la cual tuvo una fuerte repercusión en la economía de los profesionistas odontólogos ^25^. Algunos estudios señalan que la inseguridad laboral se encuentra asociada con una mayor percepción del estrés percibido ^26^^,^^27^, por lo que se resalta la importancia de mejorar este tipo de situaciones por parte de las autoridades sanitarias ^28^.

Los resultados sugieren un alto agotamiento emocional, es decir, una situación en que la fuerza o el capital emocional se consumen y el profesional siente cómo se vacía su capacidad de entrega a los demás, tanto en el nivel personal como en el psicológico; una baja despersonalización, una reacción negativa, insensible o excesivamente desconectada del individuo frente al trabajo; es la tendencia a evaluarse negativamente, con la consecuente afectación de sus habilidades para realizar su trabajo.

Se propone que las instituciones de salud consideren prevenir el SB mediante programas dirigidos a mejorar el ambiente y la cultura organizacional en los espacios laborales. Se sugiere la socialización anticipatoria, con el objetivo de acercar a los profesionales a la realidad laboral y evitar el choque con sus expectativas irreales. Se recomienda el desarrollo de los procesos de retroinformación grupal e interpersonal entre los compañeros de trabajo ^29^.

Las autoridades sanitarias de la región deberán implementar medidas de salud mental que ayuden a los profesionistas en el control del *burnout*. Asimismo, se abre la puerta para la búsqueda de determinantes asociados con las cifras del evento y que aporten un mejor entendimiento de la temática. Será importante seguir estrategias que fomenten una buena atmósfera laboral, el equilibrio de áreas vitales y una formación continua en jornada laboral, las cuales permiten modificar pensamientos ^30^^,^^31^.

La muestra del estudio seleccionada por conveniencia limita la extrapolación de los resultados en todos los odontólogos de la Jurisdicción Sanitaria 07 de la región; sin embargo, debido a la variabilidad de la población, no hay razones fundamentales que impidan comparaciones con profesionistas de sitios similares. Este estudio da pie al desarrollo de investigaciones que lleven a cabo un seguimiento de la temática con otro tipo de diseño epidemiológico que permita establecer causalidad y pueda ser representativo de la región. 

## CONCLUSIÓN

Las autoridades de salud deberán realizar estrategias de intervención organizacional centradas en reducir las situaciones generadoras del SB. Deben ejecutarse acciones que mejoren las condiciones de estabilidad laboral en los odontólogos, a través del reconocimiento de su trabajo para mejorar los tipos de contratación en las instituciones de salud del ámbito público. A su vez, el personal odontólogo deberá aprender a redistribuir su carga de trabajo, renovar su espacio, monitorear su estado emocional, regular ciclos de sueño para descansar y disfrutar los tiempos independientes en actividades recreativas, con el fin de disminuir las cifras del SB severo. 

## Fuente de financiamiento:

Ninguno

## References

[1] World Health Organization (2019). Burn-out an "occupational phenomenon": International Classification of Diseases.

[2] Maslasch C, Jackson S, Leiter MP (1996). Maslasch Burnout Inventory Manual..

[3] Cherniss C (1980). Professional Burnout in Human Service Organizations.

[4] Universidad Autónoma de Nuevo León (2018). Síndrome de burnout entre los profesionistas mexicanos.

[5] Falgueras-Vilá M, Cruzate-Muñoz C, Orfila-Pernas F, Creixell-Sureda J, González-López MP (2015). Burnout y trabajo en equipo en los profesionales de Atención Primaria. Aten. Primaria.

[6] Véliz-Burgos AL, Dörner-Paris AP, Soto-Salcedo AG, Arriagada-Arriagada A (2018). Bienestar psicológico y burnout en profesionales de atención primaria de salud en la región de Los Lagos, Chile. Acta Univ.

[7] Moro JDS, Soares JP, Massignan C, Oliveira LB, Ribeiro DM, Cardoso M (2022). Burnout Syndrome among dentists a systematic review and meta-analysis. J Evid Based Dent Pract.

[8] Bazalar M, Balarezo G (2016). El síndrome de burnout en los profesionales de odontología. Paideia XXI.

[9] Avramova N (2023). Self-perceived sources of stress and burnout determinants in dentistry - A systematic review. Galician Med J.

[10] Özarslan M, Caliskan S (2021). Attitudes and predictive factors of psychological distress and occupational burnout among dentists during COVID-19 pandemic in Turkey. Curr Psychol.

[11] Escobar-Pérez J, Cuervo-Martínez A (2008). Validez de contenido y juicio de expertos una aproximación a su utilización. Avances en Medición.

[12] Juárez-Medel CA, Hernández-Clemente J, Gutiérrez-Ventura E (2022). Factors associated with severe permanent first molar caries among adolescents from Acapulco, Guerrero. Rev Estomatol Herediana.

[13] Forné C, Yuguero O (2022). Factor structure of the Maslach Burnout Inventory Human Services Survey in Spanish urgency healthcare personnel a cross-sectional study. BMC Med Educ.

[14] Oviedo C, Campo-Arias A (2005). Aproximación al uso del coeficiente alfa de Cronbach. Rev. Colomb Psiquiatr.

[15] Zavala-González MA, Posada-Arévalo SE, Jiménez-Mayo O, López-Méndez RL, Pedrero-Ramírez LG, Pérez-Arias MB (2011). Síndrome de burnout en personal médico y de enfermería de una unidad médica familiar en Tabasco, México. Rev Med UV.

[16] IBM Corp (2017). IBM SPSS Statistics for Windows.Armonk,.

[17] Harris JK (2021). Primer on binary logistic regression. Fam Med Community Health.

[18] Khikmah K, Indahwati I, Fitrianto A, Erfiani E, Amelia R (2022). Regresión logística binaria por pasos hacia atrás para determinar el factor de tasa de crecimiento de la población en la isla de Java. Revista Jambura de Matemáticas.

[19] Orozco C, Noroña D, Vega V (2021). Síndrome de burnout en odontólogos del Seguro Social Campesino de Chimborazo en el año 2020. Scientific.

[20] Ogdon Lebrón MA, Díaz-Reissner CV (2017). Síndrome de burnout en odontólogos de la XVIII Región Sanitaria del Ministerio de Salud Pública y Bienestar Social, Paraguay. Rev Salud Pública Parag.

[21] Navarro-Guitart M, Morelatto RA (2020). Síndrome de burnout en odontólogos de la ciudad de Córdoba Rev. Salud Pública.

[22] Bravo-Pérez M, Anaya-Aguilar C, Anaya-Aguilar R, Barrios-Rodríguez R, Montero-Martín J (2019). Crisis económica y síndrome de burnout en dentistas privados en España. RCOE.

[23] Bedoya A, Díaz T, Dongo D, Guillinta G, Moncada P (2008). Síndrome de burnout en cirujanos dentistas. Kiru.

[24] Arias-Gallegos WL, Muñoz del Carpio TA, Delgado-Montesinos Y, Ortiz-Puma M, Quispe-Villanueva M (2017). Síndrome de burnout en personal de salud de la ciudad de Arequipa (Perú). Med Segur Trab.

[25] Cázares-de León F, Peraldi-Sada MG, Aneyba-López LD, Soto-Gámez DE (2021). Impacto económico en el medio odontológico durante la pandemia del COVID-19 revisión integradora. Rev ADM.

[26] Mamani-Benito O, Tito-Betancur M, Armada J, Monteza GL, Mejia CR (2022). Estrés financiero según la percepción de poder perder el trabajo en el Perú durante la pandemia COVID-19. Rev Asoc Esp Espec Med Trab.

[27] Ayacho-Palma E, Mamani-Benito OJ (2021). Inseguridad laboral y estrés percibido durante la pandemia de COVID-19 en trabajadores de una corporación privada peruana Rev. Psicol. (Arequipa. Univ. Catól. Rev. Psicol. (Arequipa. Univ. Catól San Pablo).

[28] Tirado-Montilla I, Borges-Osorio M, Mireles-Inojosa J (2017). Salud y bienestar laboral en odontólogos que trabajan en instituciones públicas de salud. Estado Carabobo. Venezuela. Salud Trab.

[29] Durán S, García J, Parra A, García M, Hernández-Sánchez I (2018). Estrategias para disminuir el síndrome de burnout en personal que labora en instituciones de salud en Barranquilla. Cultura, Educación y Sociedad.

[30] Saborío-Morales L, Hidalgo-Murillo LF (2015). Síndrome de burnout. Med Leg Costa Rica.

[31] Quezada S (2021). Síndrome de burnout: cómo prevenir problemas de salud mental en la práctica odontológica.

